# Canonical Transient Receptor Potential (TRPC) Channels in Nociception and Pathological Pain

**DOI:** 10.1155/2020/3764193

**Published:** 2020-03-21

**Authors:** Zhi-Chuan Sun, Sui-Bin Ma, Wen-Guang Chu, Dong Jia, Ceng Luo

**Affiliations:** ^1^Department of Neurosurgery, Tangdu Hospital, Fourth Military Medical University, Xi'an 710038, China; ^2^Department of Neurobiology, School of Basic Medicine, Fourth Military Medical University, Xi'an 710032, China

## Abstract

Chronic pathological pain is one of the most intractable clinical problems faced by clinicians and can be devastating for patients. Despite much progress we have made in understanding chronic pain in the last decades, its underlying mechanisms remain elusive. It is assumed that abnormal increase of calcium levels in the cells is a key determinant in the transition from acute to chronic pain. Exploring molecular players mediating Ca^2+^ entry into cells and molecular mechanisms underlying activity-dependent changes in Ca^2+^ signaling in the somatosensory pain pathway is therefore helpful towards understanding the development of chronic, pathological pain. Canonical transient receptor potential (TRPC) channels form a subfamily of nonselective cation channels, which permit the permeability of Ca^2+^ and Na+ into the cells. Initiation of Ca^2+^ entry pathways by these channels triggers the development of many physiological and pathological functions. In this review, we will focus on the functional implication of TRPC channels in nociception with the elucidation of their role in the detection of external stimuli and nociceptive hypersensitivity.

## 1. Introduction

Chronic pathological pain represents a major challenge to clinical practice and basic science. Activity-dependent neural plasticity is assumed to be a prime mechanism underlying various physiological and pathological processes including clinical transitions from acute, physiological pain to chronic, pathological pain [1, 2]. Accumulating evidence has revealed that the second messenger Ca^2+^ and Ca^2+^-dependent pathways play a crucial role in the neural plasticity, i.e., peripheral and central sensitization associated with pathological pain. Mobilization of intracellular Ca^2+^ upon neuronal activation is the main trigger for activation of a variety of signaling mediators, such as CamKII-alpha, Protein Kinase A, and extracellular receptor-activated kinases (ERK1/2); these, in turn, regulate the expression and functions of downstream proteins determining the excitability of neurons, which are involved in pain processing [1, 2]. Exploring molecular players mediating Ca^2+^ entry into cells and molecular mechanisms underlying activity-dependent changes in Ca^2+^ signaling in the somatosensory pain pathway is therefore helpful towards understanding the development of chronic, pathological pain.

The discovery of transient receptor potential (TRP) channels during the last 5 decades has increased impressively our knowledge of the molecular players mediating Ca^2+^ mobilization in the cells. TRP proteins comprise nonselective cation channels that permit the permeability of Ca^2+^ and Na+ into the cells [3]. TRP channels contribute to changes in cytosolic-free Ca^2+^ concentration either by inducing Ca^2+^ influx across plasma membrane or by driving Ca^2+^ release from several organelles. Given the unique importance of Ca^2+^ and Ca^2+^-dependent signaling in the cells, it is not surprising that TRP channels and its dysfunctions are closely linked with many physiological and pathological processes, including pain and sensitization. On the basis of amino acid homology, TRP superfamily is divided into six subfamilies, TRP canonical or classical (TRPC), TRP vanilloid (TRPV), TRP melastatin (TRPM), TRP ankyrin (TRPA), TRP polycystin (TRPP), and TRP mucolipin (TRPML) [4–9]. Among which, TRPV1, TRPM8, and TRPA1 have been extensively investigated and considered to be molecular detectors for thermal and chemical stimuli that activate sensory neurons to produce acute or persistent pain [10–12]. Although TRPC subfamily was the first to be cloned among TRP genes, lacking of specific pharmacological tools targeting at TRPC subunits led to a much lagging in the exploration of a functional role of TRPC subfamily and its underlying mechanisms. With the establishment of specific TRPC subunit transgenic mouse models and discovery of selective pharmacological tools at TRPC subunits during the past few years, emerging evidence has accumulated that TRPC subfamily exert an important role in a variety of neuronal functions, including memory, motor coordination, fear, anxiety, Huntington's disease, neurite growth, and pain [13–26]. In this review, we will focus on the role of TRPC subfamily in nociception and the modulatory mechanisms of TRPC subfamily by inflammation or injury. Recent advances in the development of therapeutic strategies targeting against TRPC subfamily will also be reviewed.

## 2. Sensory Detection and Transmission in the Pain Pathway

**Figure 1:**
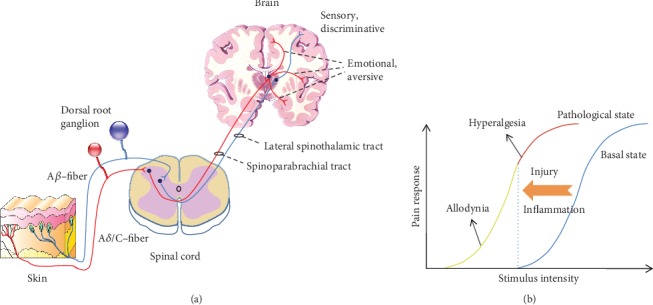
Nociceptive sensory detection and transmission in the ascending pain pathway. (a) Nociceptors detect external painful stimuli through a variety of receptors and transmit this noxious information from the periphery to the spinal cord and further central nervous system along axons, leading to sensory discrimination and affective motivation of pain. (b) Upon inflammation and injury, physiological pain converts to chronic, pathological pain, manifesting as spontaneous pain, hyperalgesia, and allodynia.

The body detects various modalities of noxious stimuli through a specialized set of sensory nociceptive fibers innervating peripheral tissues: unmyelinated C fibers and thinly myelinated A*δ* fibers, which are distinct from myelinated tactile sensors (A*β* fibers) and proprioceptors (Figure 1(a)). A variety of ion channels and receptors expressed on nociceptors, such as transient receptor potential ion channels (TRP channels), acid-sensing ion channels (ASIC channels), purinoceptor, and serotonin receptors, transduce the physicochemical properties of noxious stimuli (e.g., heat, cold, pressure, and chemicals) into electrical activity—a membrane depolarization, which is further encoded as a train of propagating action potentials by sodium channels. Nociceptive afferents carrying these peripheral signals terminate predominantly in the superficial laminae (I and II) of the spinal cord dorsal horn and form glutamatergic synapses onto second-order superficial spinal neurons, whereas nonnociceptive inputs terminate in deeper laminae (III-IV) (Figure 1(a)). The integrated nociceptive information in the superficial dorsal horn is further transmitted to projection neurons mostly located in lamina I and lamina V of the spinal dorsal horn, whose axons cross the midline and ascend to a variety of supraspinal targets. The spinal dorsal horn is therefore the site of the first synapse and processing center in the ascending pathway that conveys incoming pain information from the periphery to the central nervous system (CNS). Several pathways are demonstrated to carry the net output from spinal networks to distinct projection regions in the brain with one or several relay stations in their way so that pain is ultimately perceived in its multiple dimensions. For instance, the lateral spinothalamic tract projects multimodal sensory inputs from spinal wide-dynamic range (WDR) neurons to the lateral thalamus, the neurons from which in turn send sensory signals to the parietal somatosensory cortex. Important synapses are formed serially in the thalamus and somatosensory cortex along this ascending pathway, which has been implicated in processing sensory and discriminative aspects of pain. By contrast, the medial spinothalamic tract and the spinoparabrachial tract as well as the spinomesencephalic tract project nociceptive signals to the medial thalamus and limbic structures via monosynaptic or polysynaptic relay. From the medial thalamus, sensory inputs are further projected to widespread forebrain regions such as the anterior cingulate cortex (ACC), amygdala, and insular cortex, which are believed to mediate the emotional and aversive components of pain. The perceiving of pain in the cortex accordingly initiates information to the spinal cord and enables withdrawal reflex from the noxious stimulus (Figure 1(a)).

## 3. Transition from Acute Pain to Chronic Pain

Acute, physiological pain serves as an alarming system in our body and is characterized by a high threshold and is typically transient. However, tissue/nerve injury or inflammation often causes plastic changes of primary sensory neurons and synaptic transmission in the central nervous system, shifting physiological pain to chronic, pathological pain, which is characterized by a lowered threshold and persistent pain hypersensitivity. This includes spontaneous pain (pain experience in the absence of any obvious stimulus), hyperalgesia (an increased sensitivity to noxious stimuli), and allodynia (pain in response to normally innocuous stimuli) (Figure 1(b)) [2]. In addition, chronic pain is often accompanied by aversive emotions, such as anxiety and depression [1, 27]. The incidence of chronic pain is estimated to be 20-25% worldwide. Few patients with chronic pain obtain complete relief from the drugs that are currently available, and more than half of them report inadequate relief. Therefore, to understand the underlying mechanism that contribute to the transition from acute to chronic pain is crucial to develop better drugs to manage chronic pain. Accumulating experimental and clinical evidence indicates that both peripheral and central sensitization contribute to the development of chronic pain [1, 28, 29]. Following tissue/nerve injury, release of inflammatory mediators, increased activity of transient receptor potential channels, and dysregulation of voltage-gated ion channels are some of the changes contributing to neuronal hyperexcitability in the peripheral primary sensory neurons [28, 29]. Subsequently, this peripheral hyperexcitability sends ongoing nociceptive signals to the spinal dorsal horn and further to supraspinal brain regions, where alterations of ion channel expression, microglia and astrocyte activation, long-term potentiation of synaptic transmission, dendritic spine remodelling, and impaired descending pain modulation are among the typical changes in the central nervous system [1]. Substrates that contribute to peripheral and central sensitization are therefore potential targets for treatment of chronic pain.

## 4. TRPC Subfamily Expression in the Pain Pathway

### 4.1. Structure and Composition of TRPC Subfamily

**Figure 2:**
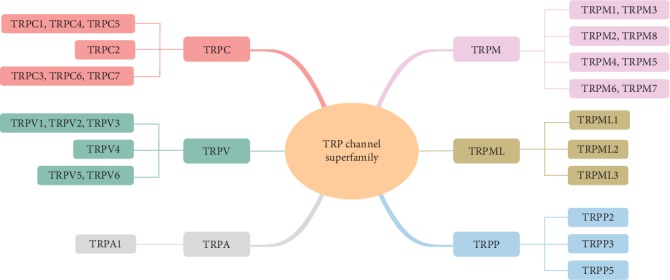
Composition of TRP channel superfamily and different TRP channel subfamily. TRP channel superfamily consists of six subfamilies, TRP canonical or classical (TRPC, 7 subunits), TRP vanilloid (TRPV, 6 subunits), TRP melastatin (TRPM, 8 subunits), TRP ankyrin (TRPA, 1 subunit), TRP polycystin (TRPP, 3 subunits), and TRP mucolipin (TRPML, 3 subunits). Based on amino acid sequence homology and functional similarities, TRPC subfamily members can be grouped into three major groups: TRPC1/4/5, TRPC2, and TRPC3/6/7.

TRP channel superfamily consists of six transmembrane domains, termed S1-S6, with cytoplasmic N- and C-terminal regions and the pore region formed by S5 and S6 segments [5, 30]. On the basis of amino acid homology, TRP superfamily is divided into six subfamilies, i.e., TRPC (TRPC1-7), TRPV (TRPV1-6), TRPM (TRPM1-8), TRPA (TRPA1), TRPP (TRPP2, TRPP3, and TRPP5), and TRPML (TRPML1-3) [4–9] (Figure 2). The first subfamily of TRP gene cloned in mammals was TRPC subfamily [31]. So far, seven members of the TRPC subfamily have been identified, i.e., TRPC1-TRPC7. Seven members of the TRPC subfamily are structurally related to Drosophila TRP but also to each other (>30% within the first 750–900 amino acids) and differ mainly within the carboxyterminal region. Based on amino acid sequence homology and functional similarities, TRPC subfamily members can be grouped into three major groups: TRPC1/4/5, TRPC2, and TRPC3/6/7 [4, 32–37] (Figure 2). TRPC2 is a pseudogene in humans, which is related but is clearly distinct from the other two major groups [31]. TRPC1, TRPC4, and TRPC5 is believed to cluster together (TRPC1/4/5) to form homo- or heteromeric channels. TRPC4 and TRPC5 are each able to form homomeric channels with similar biophysical characteristics to each other [33, 34]. In contrast, TRPC1 does not or poorly form homomeric channels, yet it readily forms heteromers with TRPC4 and TRPC5, generating ion channels with distinct biophysical characteristics [30, 38]. TRPC3, TRPC6, and TRPC7 cluster on the phylogenic tree and can form either homotetramers or heterotetramers with variable calcium ion permeability [33, 34, 39]. In aggregate, these previous studies suggest that heteromeric interactions mainly occur within members of two groups of TRPCs: TRPC1/TRPC4/TRPC5 and TRPC3/TRPC3/TRPC7. However, exceptions to these findings have also been reported. For example, exogenously and endogenously expressed TRPC1 and TRPC3 have been found to coassemble to form a heteromeric complex [40, 41].

### 4.2. Dorsal Root Ganglion: The Forefront of Pain Pathway

Dorsal root ganglion (DRG) is an aggregate of the somata of primary sensory neurons, which play an essential role in initiating somatosensation by detecting sensory stimuli in the periphery via their peripheral terminals and sending signals to the spinal cord via their central terminals [42–44]. DRG neurons are highly diverse in terms of cell sizes, gene expression, myelination levels, etc. Small-diameter neurons are assumed to be pain-sensing neurons, while medium- to large-diameter neurons preferentially detect low-threshold, nonpainful mechanical stimulation [45, 46]. Tissue inflammation or nerve injury can sensitize DRG neurons, causing exaggerated pain sensitivity, often leading to the development of chronic pain. TRP channels have been shown to be expressed in DRG neurons and to be particularly important for sensory physiology. These include roles of TRP channels for thermosensation, chemosensation, mechanosensation, tactile sensation, and hearing.

In the somatic sensory DRG neurons, TRPV1 is mainly expressed in small- to medium-diameter neurons and is sensitive to noxious heat, protons, and irritant vanilloids [10, 47, 48]. TRPV1 marks a population of unmyelinated, slowly conducting neurons (C-fibers) and account for 30-50% of all DRG neurons within rodent sensory ganglia [49, 50]. TRPM8 is activated by cold stimuli as well as a variety of natural and synthetic cooling agents, such as menthol [51, 52]. TRPM8 is mostly expressed in small-diameter, unmyelinated C-fibers as well as a minor cohort of lightly myelinated A*δ*-fibers, which constitutes 15% of all somatosensory neurons. TRPA1 serves as a detector for chemical irritants, such as isothiocyanates and thiosulfinates that constitute pungent agents from mustard and allium plants [53–56]. TRPA1 is expressed exclusively by peptidergic C-fibers [49, 53] and plays a key role in chemonociception.

### 4.3. TRPC Protein Expression in the DRG

The expression of TRPC channels was mapped in the sensory neurons of rodents throughout an embryonic and postnatal development period. Various subtypes of TRPC channels are expressed in rat and mouse primary sensory neurons [24, 57–63]. In adult mice and rat, mRNAs for TRPC1-TRPC7 were present in the DRG, with TRPC1, TRPC3, and TRPC6 being the most predominant, TRPC2, TRPC4, and TRPC5 at low levels, and TRPC7 at extremely low levels. In the embryonic stage, TRPC2 mRNAs were expressed at high levels at embryonic (E) day 12 and E14 but greatly reduced to a quite low level in adult. In contrast, TPRC1, TRPC3, and TRPC6 expression levels increased progressively from E12 to adult. TRPC4, C5, and C7 expressions increased from E12 to peak levels at E18. TRPC1 and C2 were expressed in the neurofilament-200- (NF-200-) positive large-diameter neurons, while TRPC3 mRNA expression was almost exclusively present in nonpeptidergic isolectin B4- (IB4-) positive small size neurons that were largely TRPV1-negative, which stained up to 35% of DRG neurons. A strong expression of TRPC3 in DRG was also reported by Kunert-Keil et al. [64]. In addition, TRPC5 has been found to be localized to small and medium diameter sensory neurons [65]. A single cell RNA sequencing study also determined a subset of DRG neurons express TRPC subunits [66, 67]. Interestingly, the majority of the TRPC3-positive neurons did not show TRPV1 immunoreactivity [57]. The expression and regulation of TRPC channel mRNAs in the NG were unexpectedly similar to the DRG [57]. In comparison with the well-reported expression in DRG, the profile of TRPC proteins in the spinal cord and supraspinal regions which are involved in the pain pathway has been rarely studied. In addition, in human tissue, a recent study reported differential regulation of TRPC channel gene and protein expression by intervertebral disc (IVD) degeneration and back pain in human [68]. TRPC1, C3, C4, and C6 was observed to be expressed at mRNA and protein levels in the intervertebral disc. In the degenerated IVD, TRPC6 displayed enhanced gene and protein expressions as compared to nondegenerated IVDs, while TRPC1, C3, and C4 did not display difference between degenerated and nondegenerated IVD. This study has for the first time linked TRPC6 to the degeneration of IVD and development of low back pain in human. The thorough and detailed investigation of TRPC expression in the human pain pathway remains to be further conducted.

### 4.4. Sensitivity of TRPC Proteins to Mechanical Stimuli

Emerging evidence shows that TRPC proteins confer sensitivity to mechanical stimulation in the vertebrate cells. TRPC3 and TRPC6 have been shown to be essential for normal mechanotransduction in subsets of sensory neurons and cochlear hair cells [61]. TRPC3/TRPC6 double knockout mice caused deficits in light touch and silenced half of small-diameter sensory neurons expressing mechanically activated rapidly adapting mechanosensitive currents although TRPC3 or TRPC6 single knockout mice produced no behavioral phenotype. This indicates that TPRC3 and TRPC6 may show some compensation in function, consistent with their functional redundancy observed also in other system [69]. Similarly, double TRPC3/TRPC6 knockout mice also showed hearing impairment, vestibular deficits, and defective auditory brain stem responses to high frequency sounds. In addition, another study reported that TRPC1 mutant animals display a decrease in sensitivity to innocuous mechanical stimuli and show a reduction in down-hair A*δ* and slowly adapting A*δ* fiber firing in response to innocuous mechanical stimulation [70]. TRPC1 and TRPC5 are shown to be sensitive to osmotically induced membrane stretch in cultured DRG neurons and HEK293 cells, respectively [71, 72]. TRPC6 is also activated by membrane stretch while both TRPC5 and TRPC6 activities are blocked by a tarantula toxin known to inhibit mechanosensitive channels [73]. However, it should be noted that although several TRPC channel members, including TRPC1 and TRPC6, have been proposed to contribute to mechanical sensitivity in the vertebrate cells, the functional expression of these subunits in heterologous systems remains problematic, manifesting as no alteration of mechanosensitive current by overexpression of these subunits [74]. Given this background, it is hard to see TRPC channels act as a direct mechanosensor. It is possible that TRPC proteins are necessary for regulating intracellular calcium levels that may modulate the activity of other direct mechanosensors, for example, Piezo, TMC, TACAN, or other proteins [75–78]. This possibility remains to be further explored.

## 5. Functional Roles of TRPC Subfamily in the Nociception

### 5.1. Plastic Changes of TRPC Proteins under Pathological States

Following nerve injury or tissue inflammation, TRPC proteins displayed different change profile in the DRG. Staaf et al. reported that TRPC1-TRPC7 displayed different change profile in the DRG [79]. TRPC1 and TRPC6 were not differentially regulated throughout the test period after spared nerve injury (SNI). TRPC3 was downregulated at 4 days and back to normal at 15 days and 3 months after SNI. TRPC4 had reduced expression levels at all time points after SNI while TRPC5 was downregulated at 15 days and 3 months but less reduced at 4 days. TRPC2 and C7 were not detected. In contrast, in another study with nerve injury, TRPC4 was shown to be increased at both transcriptional and protein levels in the DRG [59]. In the synovia from animal and human inflammatory arthritis, TRPC5 expression demonstrated a reduction at mRNA level, while the other TRPC proteins were not altered [26]. Repeated cyclophosphamide injections induced marked cystitis and bladder hyperactivity, which is accompanied by a specific increase in the expression of TRPC1 and TRPC4 in bladder innervating sensory neurons and the sprouting of sensory fibers in the bladder mucosa [80]. In addition, TRPC1, TRPC3, and TRPC6 have also been upregulated in the spinal cord in mice developed with morphine analgesic tolerance and hyperalgesia after chronic morphine exposure [81].

### 5.2. Functional Roles of TRPC Proteins in Pathological Pain

Nonselective blockade of TRPC channels with SKF-96365 was shown to inhibit spontaneous nociception and inflammatory pain hypersensitivity [82]. A previous study showed that TRPC1 and TRPC6 are coexpressed with TRPV4 in DRG neurons. Using antisense to TRPC1 and TRPC6, it has been proposed that they may act in concert to mediate mechanical hypersensitivity induced by carrageenan and paclitaxel chemotherapy [24]. Chronic pain usually accompanies immune-related disorders with an elevated level of serum IgG immune complex (IgG-IC), but the underlying mechanisms are obscure. A previous study by interfering TRPC3 with siRNA reported that TRPC3 is required for IgG immune complex-induced excitation of rat DRG neurons [60]. There is also evidence showing that TRPC4 is required for the detection and/or transmission of colonic MO visceral pain sensation [25]. Rats with a transposon-mediated TRPC4-knockout mutation displayed tolerance to visceral pain induced by colonic mustard oil (MO) exposure, but not somatic or neuropathic pain stimuli. Moreover, wild-type rats treated with a selective TRPC4 antagonist (ML-204) prior to MO exposure mimicked the behavioral responses observed in TRPC4-knockout rats.

Although the TRPC protein expression profile in the supraspinal regions responsible for pain perception is rarely investigated, a recent study by Wei et al. showed a facilitating regulation of neuropathic pain by amygdaloid TRPC4/C5 channels [23]. Chronic cannula for microinjection of ML-204, a TRPC4/C5 antagonist, produced a dose-dependent inhibition of pain hypersensitivity as well as anxiety-like behavior associated with nerve injury. This result indicates that amygdaloid TRPC4/C5 may contribute to the maintenance of pain hypersensitivity and pain-related aversion in neuropathy. In addition, spinal TRPC6 channels are shown to contribute to morphine-induced antinociceptive tolerance and hyperalgesia in rats [81]. Knockdown of TRPC6 in the spinal cord prevents the development of morphine-induced tolerance and hyperalgesia. In comparison with the pronociceptive action of other TRPC proteins, TRPC5 is recently reported to be able to protect against pain and vascular inflammation in arthritis and joint inflammation [26]. In this study, following complete Freund's adjuvant- (CFA-) induced unilateral arthritis, genetic ablation (TRPC5 KO mice) or pharmacological blockade of TRPC5 displays augmented weight-bearing asymmetry, enhanced secondary hyperalgesia, and increased cytokine concentrations in the synovial lavage fluid. This result suggests that the activation of TRPC5 may be associated with an endogenous anti-inflammatory/analgesic pathway in inflammatory joint conditions [26].

## 6. The Underlying Mechanisms for Functional Roles of TRPC Proteins in Pathological Pain

### 6.1. Regulation of Calcium Homeostasis by TRPC Proteins in Primary Sensory Neurons

It has recently been shown that an abnormal persistent increase in intracellular calcium levels mediates the transition from acute to chronic pain in inflammatory states [1, 83, 84]. Thus, the regulation of intracellular calcium level could be a key mechanism in preventing pain chronicity. TRPC channels (including subtypes 1-7), a family of Ca^2+^-permeable nonselective cation channels, play a critical role in the regulation of intracellular Ca^2+^ homeostasis and membrane excitability in excitable cells [85]. It has been thought that TRPC channels contribute to receptor-operated calcium entry (ROCE) or store-operated calcium entry (SOCE) through PIP2 hydrolysis by PLC activation. Accumulated evidence has suggested that TRPC3, TRPC6, and TRPC7 form agonist-regulated channels that can be directly activated by DAG in different biological system [86–88]. This inference is further supported by recent Cryo-EM structure analysis of TRPC3 and TRPC6 [39, 89, 90]. In contrast to good consensus regarding a direct binding of DAG to TRPC3/6/7 subfamily, there is a controversial debate on the role of TRPC channels as candidates of SOCE. Although TRPC1, TRPC4, and TRPC5 channels have been linked to SOCE in several different biological systems [91–93], none of the TRPC channels generate Ca^2+^ currents that resemble the *I*_SOC_ current [94]. Nevertheless, this does not exclude the possibility that TRPC channels also participate in SOCE under certain scenarios, such as the assembly with the STIM1-Orai1 complex, which have been identified as the key components of SOCE. There is evidence showing TRPC1 are assembled to form a dynamic STIM1-Orai1-TRPC1 ternary complex to drive the *I*_SOC_ current in human embryonic kidney (HEK) cells and human salivary gland cells [94–97]. However, both the cellular physiology of TRPC channels in primary sensory neurons and their underlying mechanisms in the regulation of nociception have yet to be clarified.

**Figure 3:**
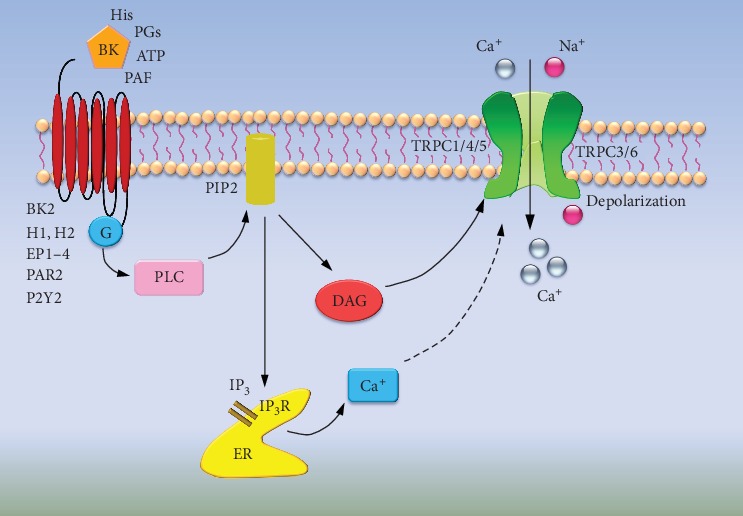
The proposed mechanisms underlying functional roles of TRPC channels in nociceptive hypersensitivity. Following inflammation or injury, a variety of proinflammatory mediators, such as bradykinin, prostaglandins, histamine, platelet-activating factor, and ATP, are released from the injured site and activates their corresponding Gq-protein-coupled receptors (GPCRs) in the peripheral nociceptors. These GPCRs couple with PLC and cleave PIP2 into DAG and IP3, involving the activation of TRPC3/6 directly by DAG, or the activation of TRPC3/6 or TRPC1/4/5 by IP3-induced depletion of endoplasmic reticulum calcium store. The resulting increase of calcium level via TRPC channels leads to depolarization of nociceptors, which in turn causes peripheral sensitization and nociceptive hypersensitivity. BK: bradykinin; His: histamine; PAF: platelet-activating factor; BK2: bradykinin receptor 2; PLC: phospholipase C; PIP2: phosphatidylinositol 4,5-bisphosphate; DAG: diacylglycerol; IP3: inositol trisphosphate.

TRPC channels integrate several types of intracellular stimuli, including PLC and PKC activity, DAG level, intracellular calcium levels, and PIP2 levels into changes in membrane potential and calcium entry [32]. Of note, TRPC proteins have been shown to be expressed in the primary sensory neurons along the somatosensory pathway which might contribute to inflammatory pain [24, 57–63]. Following inflammation or injury, the inflamed area releases various proinflammatory mediators, which forms a soup to trigger oedema, itchiness, redness, and peripheral sensitization [28, 98]. This “inflammatory soup” comprises a variety of signaling molecules, such as bradykinin, prostaglandins, histamine, platelet-activating factor, and ATP, which are able to activate their corresponding receptors located in nociceptors and cause hyperexcitability of nociceptors [99]. Many of these receptors include well-characterized Gq-protein-coupled receptors (GPCR), such as bradykinin B2, histamine receptor H1 and H2, P2Y2 purinoceptor, and protease-activated PAR2 receptor [99]. The activation of these GPCRs by agonist binding and their coupling with phospholipase C (PLC) cleaves the phosphatidylinositol 4,5-bisphosphate (PIP2) into two bioactive products, diacylglycerol (DAG), and inositol trisphosphate (IP3). Both of these two compounds act to increase intracellular Ca^2+^ levels through two distinct mechanisms: one is referred to as receptor-operated Ca^2+^ entry (ROCE), involving the activation of calcium permeable channels directly by DAG [87, 88, 99]. The other is store-operated Ca^2+^ entry (SOCE), involving the activation of calcium channels by IP3-induced depletion of endoplasmic reticulum calcium stores. Hence, these two processes via PLC activation collectively result in both intracellular Ca^2+^ increase and enhanced extracellular Ca^2+^ influx across the plasma membrane following depletion of intracellular Ca^2+^ store (Figure 3). In a recent study by Alkhani et al., the authors identified TRPC3, a highly expressed TRPC protein in DRG neurons, is coupled to several classes of proinflammatory metabotropic receptors and plays a significant role in Ca^2+^ homeostasis and sensitization in primary nociceptors, through its involvement in both SOCE and ROCE [62]. Indeed, TRPC3 has been shown to be required for the cellular response to IgG immune complex (IgG-IC), a pain-inducing inflammatory compound that binds to the GPCR Fc-gamma receptor I (Fc*γ*RI), which in turn is coupled to TRPC3 through the Syk-PLC-IP3 pathway [60]. Similarly, with the use of nonselective TRPC inhibitor SKF-96365, TRPC channels were revealed to be involved in the inflammatory component melittin-induced Ca^2+^ increase and activation of primary nociceptors [100]. These results indicate that regulation of Ca^2+^ homeostasis in the primary sensory neurons by TRPC proteins provides a cellular basis for its role in nociception.

## 7. Novel Pharmacological Tools for TRPC Proteins

TRPC channels are widely expressed in the nervous system and permit the influx of Na^+^ and Ca^2+^ ions into the cells. The establishment of transgenic mouse models of different subtypes of TRPC channels and the development of subtype-specific pharmacological modulators for TRPC channels are crucial for the distinction of respective TRPC channels that play particular physiological and pathological roles in the native system. With these important tools, accumulating evidence has emerged that TRPC channels play pivotal roles in many physiological and pathological processes, including memory, cardiovascular diseases, neurological disorders, cancer, chronic kidney disease, pain, and other pathological conditions. However, it is important to point out that many of previous research on TRPC channels were based on genetic evidence through which phenotype was examined in animals or cells with naturally occurring mutations or gene knockout or knockdown. Given the apparent structural and functional redundancy observed in TRPC subunits, the genetic-based approaches may suffer from compensatory changes between subunits, which may mask the function of specific TRPC subunit under some circumstances. In recent years, there has been a great progress in the exploration of selective and specific compounds acting on different subtypes of TRPC channels. Here, we summarize several characterized important pharmacological tools to specifically activate or block TRPC channel subtype, with which for further exploration of the functional significance and translational potential of TRPC channels.

### 7.1. Broad Range TRPC Channel Blockers

#### 7.1.1. SKF-96365

Organic synthetic blockers have been recognized to interfere with receptor-dependent and store-operated calcium entry mechanisms [101, 102]. SKF-96365, 1-{*β*-[3-(4-methoxyphenyl) propoxy]-4-methoxyphenethyl}-1H-imidazole hydrochloride, is an inhibitor of receptor-mediated as well as store-operated calcium entry mechanisms [101, 102]. The molecular identification, cloning, and functional characterization of a great variety of ion channels enabled the identification of SKF-96365 as a broad range TRPC channel blocker. In rats with inflammatory pain induced by melittin injection, localized peripheral administration of SKF-96365 prevented the development and maintenance of spontaneous nociception and hyperalgesia [82]. Further mechanistic analysis revealed that SKF-96365-induced antinociception was mediated by inhibition of melittin-evoked calcium increase and hyperexcitability in primary sensory neurons [100].

### 7.2. Selective Modulators of TRPC3/6/7 Channels

#### 7.2.1. Hyperforin

Hyperforin, a bicyclic polyprenylated acylphloroglucinol derivative, is the main active principle of St. John's wort extract responsible for its antidepressive profile. In contrast to classic, synthetic antidepressants, hyperforin reduces monoamine uptake by elevating the intracellular sodium concentration and subsequent elimination of the sodium gradient as required for neurotransmitter transporter function [103, 104]. The attempts to identify the molecular target of hyperforin by Harteneck and Gollasch led to the identification of TRPC6 [105]. Further studies revealed that hyperforin-induced cation entry was highly specific and related to TRPC6 and was suppressed in cells expressing a dominant negative mutant of TRPC6, whereas phylogenetically related channels, i.e., TRPC3, remained unaffected. The discovery of hyperforin as a selective TRPC6 channel activator is exciting because modulation of TRP channels by secondary plant compounds is not restricted to TRPA, TRPV, or TRPM subfamilies [105]. These data provided an important pharmacological tool to investigate TRPC6 function.

#### 7.2.2. GSK1702934A and Structural Analogs

Additionally, a small 1,3-dihydro-2H-benzo [d]imidazol-2-one-based potent agonist, GSK1702934A, has been identified as a tool to directly activate TRPC3/6 channels. In HEK293 cells expressing human TRPC3 and TRPC6, GSK1702934A was shown to induce a current with EC50 at 80 and 440 nM [106, 107]. Based on the main scaffold of GSK1702934A, an azobenzene photoswitch moiety was added to produce a light-sensitive TRPC agonist, OptoBI-1, which possesses the ability to preferentially activate TRPC3/6/7, but not TRPC4/5 [108]. It has been reported that light stimulation can suppress the action potential firing induced by current injection in hippocampal neurons exposed to OptoBI-1. In addition, a recent study synthesized a positive allosteric TRPC6 modulator, namely, C20 [109]. This compound shows higher selectivity at TRPC6 over closely related TRPC3 and TRPC7.

#### 7.2.3. Pyrazole Derivatives

Kiyonaka et al. identifies a pyrazole compound (Pyr3) as a selective TRPC3 inhibitor [110]. The direct action of Pyr3 on the TRPC3 protein was demonstrated by electrophysiological and photoaffinity-labeling experiments. Moreover, Pyr3 was shown to be capable of eliminating B cell receptor-induced Ca^2+^ oscillation regulated by TRPC3-mediated Ca^2+^ influx and attenuating activation of nuclear factor of activated T cells as well as hypertrophic growth in cardiomyocytes. These findings are certainly exciting because Pyr3 enables a pharmacological dissection of closely related TRPC subtypes and provides a powerful tool to study *in vivo* function of TRPC3. Previous studies reported the requirement of TRPC3 for IgG immune complex-induced excitation of rat DRG neurons under chronic pain states. A pyrazole compound, Pyr3, was shown to suppress the neuronal excitation induced by IgG immune complex in DRG [60]. However, later investigations questioned on the selectivity of pyrazole inhibitors and demonstrated Pyr3 inhibit STIM/Orai Ca^2+^ entry complexes [111]. Schleifer et al. identified other pyrazole derivatives, especially Pyr10, as a selective TRPC3 blocker, which is able to distinguish between TRPC3 and Orai-mediated SOCE. Pyr10 showed much more potency on TRPC3 than Orai-mediated SOCE [111]. A previous study demonstrated that TRPC3 channels contribute to SOCE and inflammatory transductions in primary nociceptors. Delivery of TRPC3 antagonist, Pyr10, was found to result in a substantial decrease of SOCE [62].

#### 7.2.4. GSK Compounds

GSK compounds, i.e., GSK2332255B and GSK2833503A, have been shown to selectively block the activities of TRPC3 and TRPC6 with IC_50_ at 3-21 nM [112]. These two compounds display more than 100-fold selectivity over other calcium-permeable channels and 100-fold greater potency at TRPC3 compared with Pyr3. Functional analysis revealed that GSK2332255B and GSK2833503A possess the capability to inhibit pathological cardiac hypertrophy [112].

#### 7.2.5. BI 749327

A recent study by Lin et al. developed an orally bioavailable TRPC6 antagonist, BI 749327 [113], and tested its effect in *in vivo* disease models. BI 749327 was reported to have an 85- and 42-fold selectivity on TRPC6 (IC_50_ 13 nM against mouse TRPC6, t1/2 8.5-13.5 hours) over the most closely related channels, TRPC3 and TRPC7. *In vitro* and *in vivo* data collectively supported BI 749327 as a selective pharmacological TRPC6 inhibitor with oral bioavailability and suitable pharmacokinetics to ameliorate TRPC6-mediated disorders.

#### 7.2.6. Other TRPC6 Inhibitors

Recently, SAR7334 synthesized from a series of aminoindanol derivatives was identified as a potent TRPC6 inhibitor with an IC_50_ at 7.9 nM [114]. This compound showed higher selectivity for TRPC6 over TRPC3/7 channels. In addition, another compound DS88790512, a bicyclo[4.3.0]nonane derivative, has also been demonstrated to inhibit TRPC6 with an IC_50_ at 11 nM [115]. Beyond this, some compounds derived from a natural product, i.e., SH045, larixyl acetate, have also the capability to inhibit TRPC6 channels with higher selectivity over TRPC3 and TRPC7 [109, 116].

### 7.3. Selective Modulators of TRPC1/4/5 Channels

#### 7.3.1. (-)-Englerin A

(-)-Englerin A (EA) was found to be a highly efficient, fast-acting, potent, selective, and direct stimulator of TRPC4 and TRPC5 channels [117]. EA produced the rapid and selective killing of renal cancer cells through the activation of TRPC4/5 channels. The EC50 of EA for the activation of TRPC4 channels is 11.2 nM and 7.6 nM for TRPC5 channels [117]. A recent study identified an (-)-englerin A analog, which antagonizes (-)-englerin A at TRPC1/4/5 channels [118].

#### 7.3.2. AM237

Recent research has yielded specific and potent xanthine-based TRPC1/4/5 inhibitors, such as Pico145; a newly published study by Minard et al. reported the possibility of xanthine-based activation of TRPC5 channels [119]. AM237, an analog of the TRPC1/4/5 inhibitor Pico145, was synthesized and showed the capability to activate homomeric TRPC5 channels, but not other subtypes of TRP channels. This study supports the general principle that xanthine-based small molecules can activate, potentiate, or inhibit these channels depending on subunit composition.

#### 7.3.3. Benzothiadiazine Derivative and Methylprednisolone

Recently, Beckmann *et al*. screened compound libraries and identified the glucocorticoid methylprednisolone and N-[3-(adamantan-2-yloxy)propyl]-3-(6-methyl-1,1-dioxo-2H-1*λ*6,2,4-benzothiadiazin-3-yl)propanamide (BTD) as novel TRPC5 activators [120]. These compounds exhibited selectivity for TRPC5 channels or heteromeric channel complexes containing TRPC5 but failed to activate the other TRP channel subtypes, i.e., TRPC3, TRPC6, TRPC7, TRPA1, TRPV1, TRPV2, TRPV4, TRPM2, and TRPM3.

#### 7.3.4. Tonantzitlolone

TZL is a novel potent agonist for TRPC1/4/5 channels, which shows potency at a nanomolar level [118]. TZL activated overexpressed channels with EC50 values of 123 nM (TRPC4), 83 nM (TRPC5), 140 nM (TRPC4-TRPC1), and 61 nM (TRPC5-TRPC1). These effects of TZL were potently antagonized by the TRPC1/4/5 inhibitor Pico145. TZL failed to activate endogenous store-operated Ca^2+^ entry or overexpressed TRPC3, TRPV4, or TRPM2 channels in HEK293 cells.

#### 7.3.5. Pico145

For exploration of the physiological significance and translational potential of TRPC1/4/5 channels, it is of great importance to develop pharmacological tools with which to specifically block the channels. A recent study by Rubaiy et al. characterized a potent, specific antagonist, Pico145 at TRPC1/4/5 channels [38]. The potency of Pico145 ranged from 9 to 1300 pM, depending on the TRPC1/4/5 subtype and activation mechanism. The heteromeric channels TRPC4/TRPC1 or TRPC5/TRPC1 concatemers showed more sensitivity to Pico145 with IC_50_ at 33 pM and 199 pM, respectively. Moreover, the other TRP channel subtypes were not affected by Pico145, such as TRPC3, TRPC6, TRPV1, TRPV4, TRPA1, TRPM2, TRPM8, and store-operated Ca^2+^ entry mediated by Orai1 [38, 121].

#### 7.3.6. ML204

A study by Miller et al. reported the identification and characterization of ML204 as a novel, potent inhibitor for TRPC4 and TRPC5 channels [122]. ML204 was shown to inhibit TRPC4*β*-mediated intracellular Ca^2+^ elevation with IC_50_ at 0.96 *μ*M and exhibit 19-fold selectivity against muscarinic receptor-coupled TRPC6 channel activation. In DRG neurons, ML204 at 10-20 *μ*M showed no appreciable blockade of the other TRP channel subtype, including TRPV1, TRPV3, TRPA1, TRPM8, and KCNQ2 as well as native voltage-gated Na^+^, K^+^, and Ca^2+^ channels. Of note, native TRPC1/4/5 channels exist as heteromers, but ML204 was less sensitive to heteromeric channels, such as TRPC4/TRPC1. Whole-cell patch clamp recording demonstrated that ML204 display potent inhibition on homomeric TRPC4 channels. In a rat visceral pain model induced by colonic injection of mustard oil, intraperitoneal administration of ML204 produced a prominent antinociceptive effect [25]. Moreover, microinjection of ML204 in the amygdala led to a dose-dependent pain relief without obvious side effects. Additionally, amygdaloid administration of ML204 reduced aversive emotion associated with neuropathic pain [23].

#### 7.3.7. Benzimidazoles

A recent study by Sharma *et al*. reported the synthesis and biological characterization of a series of N-heterocyclic-1-benzyl-1Hbenzo[d]imidazole-2-amines as selective TRPC5 inhibitors [123]. This team evaluated the benzimidazole scaffold and substituents resulting in the discovery of AC1903, a novel TRPC5 inhibitor that is active in multiple animal models of chronic kidney disease (CKD). This compound was shown to have an IC_50_ at 4.06 *μ*M, as determined using the SyncroPatch. An *in vivo* pharmacokinetic study demonstrated that this compound has PK values similar to ML204 and is selective for TRPC5 over TRPC4 and TRPC6. In addition, another 2-aminobenzimidzaole derivative, M084, was identified to be an TRPC4/5 inhibitor. Although not as potent as ML204, M084 has better pharmacokinetic properties and similar potency at TRPC4 and TRPC5 channels with IC_50_ at about 10 *μ*M. Intraperitoneal injection of ML084 produced a profounding antidepressant and anxiolytic effects in mice [124].

## 8. Concluding Remarks and Future Directions

Despite great progress that has been made in our understanding of the chronic pain in the last decades, chronic pain still represents a major public health problem. This mainly manifests as two unevadable facts: one, the underlying mechanism remains elusive, and two, treatment of chronic pain in clinic still faces serious challenges. TRPC channels form nonselective cation channels with higher permeability for Ca^2+^ and Na^+^ into the cells and expressed in the pain pathway, especially in the DRG—the first site for the detection of nociceptive signals [24, 28, 57–63]. Emerging evidence has indicated a potential role of TRPC channels in the detection of external stimuli and nociceptive hypersensitivity under pathological states. Nevertheless, our understanding of these ion channels in nociception and nociceptive hypersensitivity is far from being revealed. In recent years, the resolution revolution in cryoelectron microscopy brought several high-resolution structures of TRP channels. What excites us is that the electron cryomicroscopy structure of the TRPC3, TRPC6, and TRPC4 proteins has been resolved just recently [39, 89, 125, 126]. This can help to study TRPC channel pharmacology and activation mechanisms, which in turn offer the opportunity to develop more potent, selective pharmacological tools to explore the physiological and pathological functions of TRPC channels, including pain and sensitization.
